# Advanced Renal-Cell Carcinoma Pseudoprogression After Combined Immunotherapy: Case Report and Literature Review

**DOI:** 10.3389/fonc.2021.640447

**Published:** 2021-05-28

**Authors:** Xiaojie Zhang, Hao Huang, Lu Han, Tiepeng Li, Zibing Wang, Quanli Gao

**Affiliations:** Department of Immunotherapy, Affiliated Cancer Hospital of Zhengzhou University & Henan Cancer Hospital, Zhengzhou, China

**Keywords:** pseudoprogression, immunotherapy, renal-cell carcinoma, cytokine-induced killer (CIK) cells, PD-1

## Abstract

Treatment with a combination of programmed cell death-1 (PD-1) blocker and cytokine-induced killer (CIK) cells has improved outcome in cancer patients but is also associated with various patterns of responses. Pseudoprogression is a unique and uncommon phenomenon with no clear criteria for rapid diagnosis. Although some reports of pseudoprogression during immunotherapy exist, there are few reports of pseudoprogression occurring twice in the same patient. Here, we report the case of 51-year-old female patient with advanced renal cell carcinoma, who received a combination treatment of PD-1 blocker and CIK cells, and where pseudoprogression of lung and brain tumors occurred successively during treatment.

## Introduction

The incidence of kidney cancer is increasing, with renal cell carcinoma (RCC) now accounting for approximately 85% of adult kidney cancers, about 70% of which are clear cell carcinoma (1). The treatment strategy for advanced RCC is evolving from one using multiple target-kinase inhibitors to one using immunotherapy (2). When successful, immunotherapy treatment significantly prolongs overall survival in advanced RCC (3, 4), but it succeeds in only a minority of patients (4). Many clinical trials using combination immunotherapy for advanced RCC have been conducted (3), but few studies exist using immunotherapy combined with cytokine-induced killer (CIK) cells (4).

Given the unique mechanism of immunotherapy, many unconventional phenomena can appear. Pseudoprogression is one such treatment response reported in many tumors treated with immunotherapy (5). Recognizing and understanding this phenomenon is crucial since it may lead to discontinuing therapy in patients that are actually responding positively. Although pseudoprogression can be judged in many ways (6–8), it is difficult to quickly distinguish it from real progression. Here, we present a case of RCC metastasized to the lung and brain where significant pseudoprogression appeared twice during treatment with the PD-1 inhibitor nivolumab combined with CIK cells.

## Case Presentation

A lump found in the left kidney during physical examination of a 51-year-old woman was diagnosed as kidney cancer, and the woman underwent a radical nephrectomy of the left kidney *via* laparoscope surgery on 29 November 2016. She had no history of kidney disease nor any family history of cancer. Pathology revealed a nuclear grade 2 clear cell carcinoma (TNM-staging: pT2 pN0 G2 M0) with favorable prognostic features according to MSKCC criteria, and thus the patient underwent a regular comprehensive review after surgery. Right middle femur metastasis occurred in January 2018, and positron emission tomography-CT (PET-CT) showed multiple lung metastases on 5 February 2018. At this time, she experienced low fever, poor appetite, and fatigue. Physical examination of lungs showed clear breath sounds, no weakening and no rales. She began therapy with sunitinib, and zoledronic acid, 4 mg every 4 weeks. During sunitinib treatment, a grade 2 hand-foot skin reaction occurred. After a 2-month treatment, a CT showed that the lung metastasis had increased in size, which was evaluated as progressive disease according to *RECIST* 1.1.

**Figure 1:**
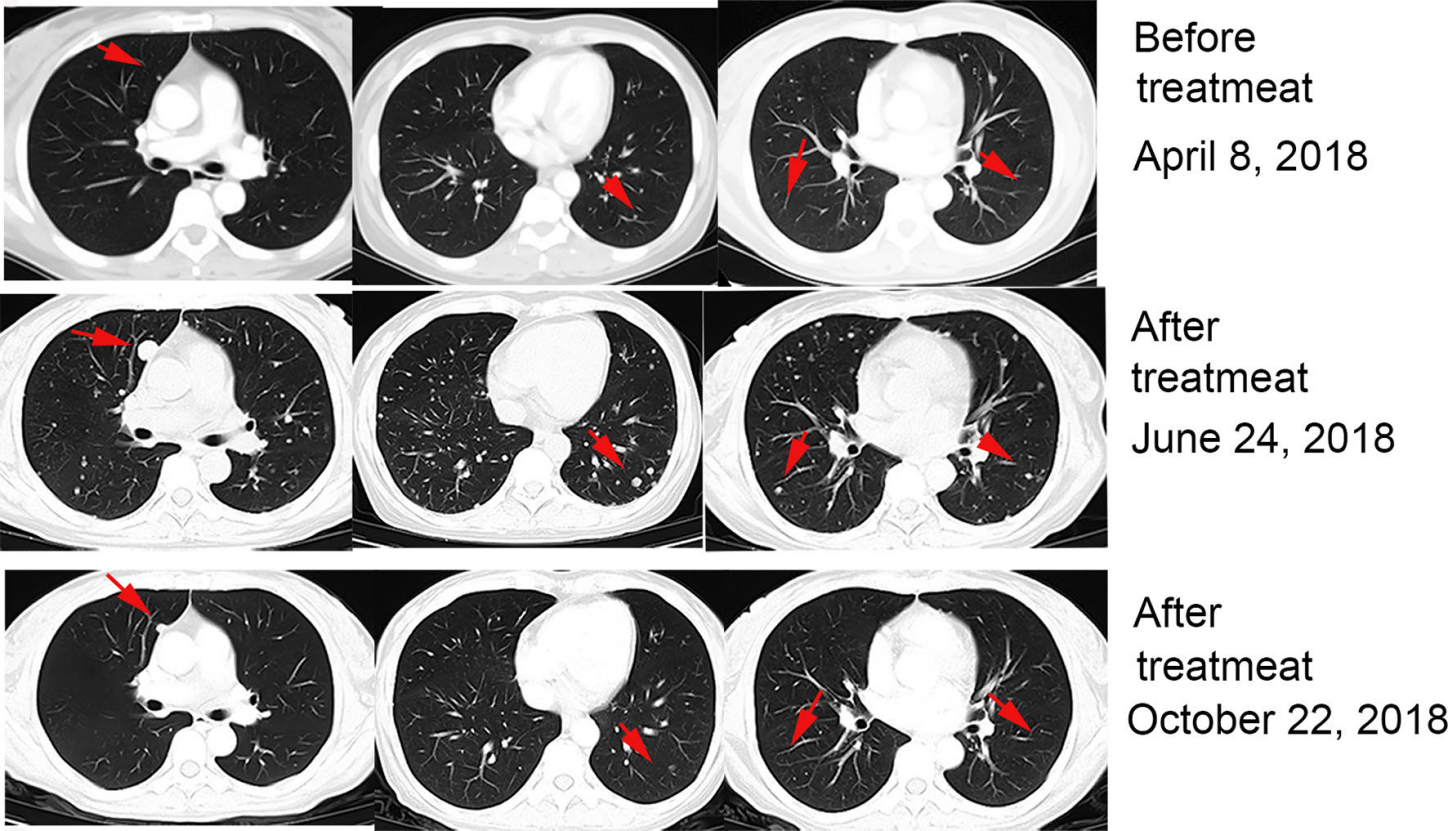
Patient exhibited pseudoprogression of pulmonary lesions after 2 months of treatment with nivolumab plus CIK cell transfer and achieved a partial remission after 4 months.

After providing informed consent, the patient received 2 mg/kg nivolumab combined with approximately 5 × 10^9^ CIK cells every 3 weeks starting in April 2018. After two cycles of therapy, the patient experienced increased appetite, but a CT scan performed 2 months after initiation of this therapy revealed enlarged and new pulmonary metastases. We continued to apply nivolumab and CIK cells combination therapy. After 6 months of therapy, a CT showed that the lesions in the lungs had begun to decrease (**Figure 1**). Subsequent CT scans showed progressive disease reduction until November 2019.

**Figure 2:**
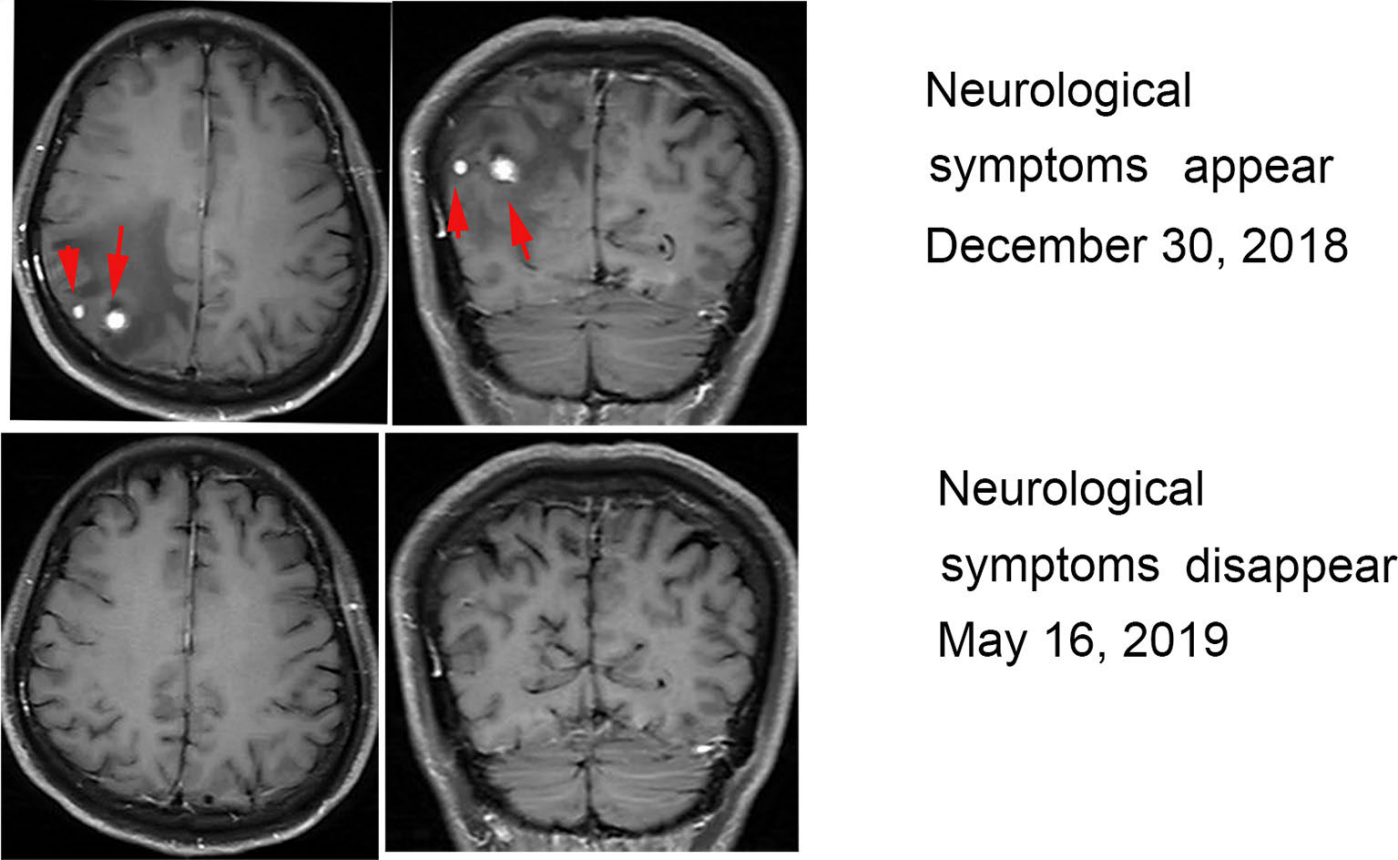
MRI during treatment with nivolumab and CIK cells. MRI revealed two enhancing metastatic lesions within the brain parenchyma 6 months after the initiation of treatment. The brain lesions disappeared after 6 months of continued treatment.

In December 2018, the patient experienced intermittent headaches. No fever, nausea or vomiting was present, and no change in blood pressure, appetite and physical status occurred. Enhanced head magnetic resonance imaging (MRI) showed two nodules on the right parietal lobe that were considered metastases. After multidisciplinary consultation, local radiotherapy for head lesions was recommended, but the patient refused. Oral painkillers were used to control headaches and treatment with nivolumab and CIK cells continued. After an additional 5 months (May 2019), symptoms disappeared, and another enhanced MRI found the brain lesions had disappeared (**Figure 2**).

During treatment with nivolumab and CIK cells, no adverse events occurred except for a fever of 38.8 °C at the beginning of therapy. This was managed with physical cooling treatment; no corticosteroids were given.

**Figure 3:**
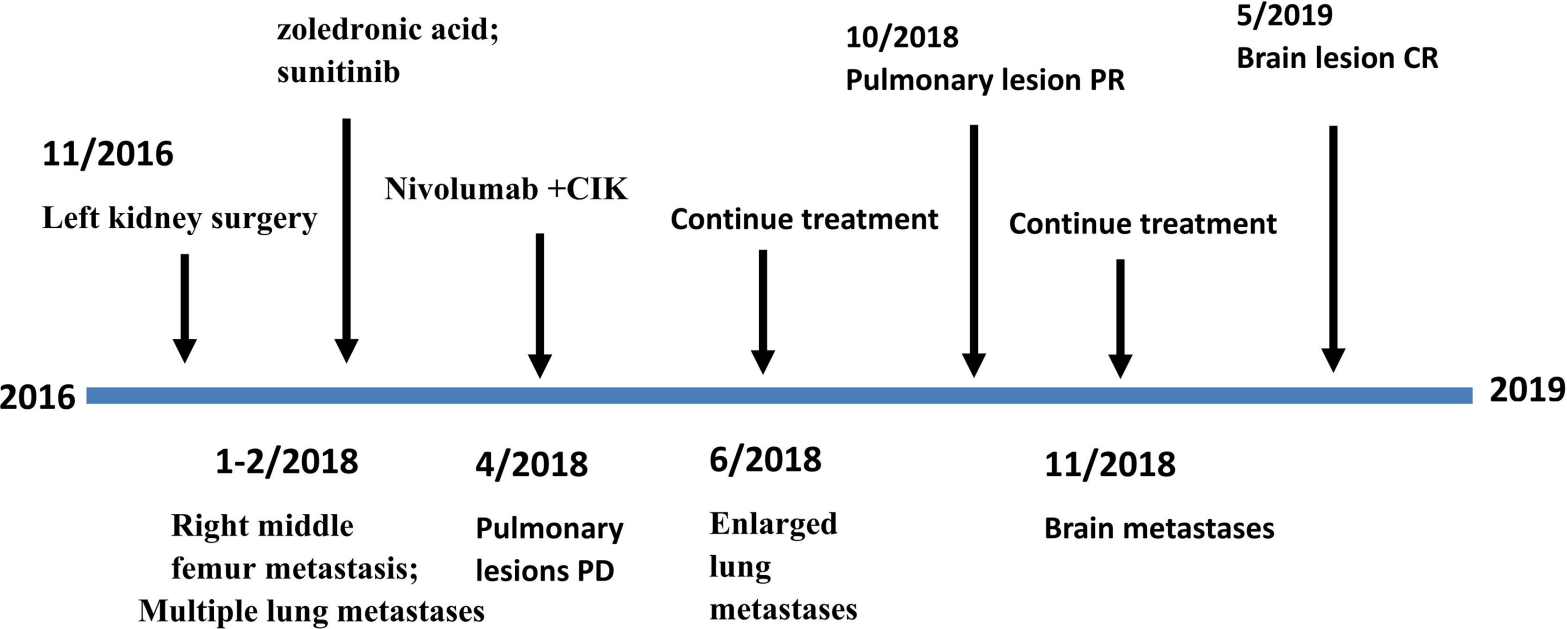
Timeline of the patient treatment.

At a follow-up in November 2019, CT scans showed right hilar lymph nodes that were significantly enlarged and low fever, fatigue, and poor appetite were present. Treatment with axitinib was started, achieving partial remission (PR). As of the last follow-up on 6 May 2020, the disease continues to stabilize. The timeline of the patient’s disease development, treatment and outcome is shown in **Figure 3**.

## Discussion

Immune checkpoint inhibitors as a new anti-tumor drug have completely changed the treatment mode for kidney cancer. However, the objective response rate is only about 25% and complete remission rate is only about 1% (9). Therefore, a combination treatment strategy is the focus of current research. Research on the combination of targeted chemotherapy drugs and combined immunotherapy is prevalent in the literature. However, few studies on the combination of targeting PD-1 and using CIK cells exist.

CIK cells are a group of heterogeneous immune-active host effector cells created from peripheral blood lymphocytes that are stimulated by interleukin-2 (IL-2), anti-CD3 antibodies, and interleukin-1 (IL-1) *in vitro*. CIK cells express the dual-functionality of acquiring NK cell function and retaining TCR-mediated cytotoxicity with T cell subsets (10). Blockade of the PD-1 pathway in combination with CIK cells as a treatment program shows better survival advantage and fewer adverse reactions in RCC and lung cancer (4), although the precise mechanisms of this are not clear. One possible mechanism is be that anti-PD-1 therapy increases the number of effector T cells in the tumor microenvironment, and CIK cells, essentially T lymphocytes, have a direct cytotoxic effect against tumor cells (10). Previous studies have shown that CIK cells improve quality of life and progression-free survival rates in patients with cancer (11). In addition, CIK cells alone, or combined with anti-PD-1 therapy, have no significant side effects in clinical applications (12, 13). This may thus provide a new treatment options for some patients who have poor physical fitness scores and cannot tolerate other treatments. However, only a few cases of this treatment have been reported (13), and further research is needed to verify these findings.

Pseudoprogression is a special phenomenon that occurs during immunotherapy. It is defined as the presence of a new lesion or an increase in size of an existing lesion, followed by a decrease in the tumor burden. It thus appears to be disease progression until the next imaging assessment. Biopsies have confirmed it is essentially an influx of inflammatory cells, necrosis, or sarcoid-like reactions. The overall incidence of pseudoprogression in solid tumors is low. If pseudoprogression occurs and the patient is clinically stable, according to iRECIST, the patient can continue treatment. However, some patients may consider changing treatment options for fear they have true disease progression. Thus, distinguishing between true progression and pseudoprogression is especially important. iRECIST criteria are currently the definitive guidelines for measuring objective response rates in cancer immunotherapy trials. According to iRECIST, waiting for 4-8 weeks until the next imaging assessment should occur before changing treatment (6). In our case, when lung lesions progressed for the first time and new lesions appeared in the brain, without deterioration in clinical symptoms, we did not change the treatment plan. Subsequent imaging examination confirmed that the change was pseudoprogression. However, patients with true disease progression will have delayed potentially effective treatment. Studies suggest changes in serum interleukin-8 (IL-8) levels and circulating tumor DNA may be useful in the diagnosis of pseudoprogression (7, 8).

Our case demonstrates that pseudoprogression can occur at any time during immunotherapy and that clinicians need to pay special attention to identifying it. Characterizing the relationship between pseudoprogression and long-term survival requires longer term, large sample size observations.

## Data Availability Statement

The original contributions presented in the study are included in the article/supplementary material. Further inquiries can be directed to the corresponding authors.

## Ethics Statement

The study was conducted in accordance with the principles of the Declaration of Helsinki, and the study protocol was approved by the ethics committee of Henan Cancer Hospital. We obtained patient consent before the study. Written informed consent was obtained from the participant for the publication of this case report. Manuscript is approved by all authors for publication.

## Author Contributions

XZ collected data and wrote the manuscript. ZW and QG appraised and edited the manuscript. HH and LH provided images for publication. All authors contributed to the article and approved the submitted version.

## Funding

National Natural Science Foundation of China (81972690, 81272526) and Science and Technology Department of Henan Province (51010205-206499).

## Conflict of Interest

The authors declare that the research was conducted in the absence of any commercial or financial relationships that could be construed as a potential conflict of interest.

## Abbreviations

PD-1, programmed cell death-1; RCC, renal cell carcinoma; CIK, cytokine-induced killer; PET-CT, positron emission tomography-CT.
